# Anti-corrosive and oil sensitive coatings based on epoxy/polyaniline/magnetite-clay composites through diazonium interfacial chemistry

**DOI:** 10.1038/s41598-018-31508-0

**Published:** 2018-09-06

**Authors:** Khouloud Jlassi, A. Bahgat Radwan, Kishor Kumar Sadasivuni, Miroslav Mrlik, Aboubakr M. Abdullah, Mohamed M. Chehimi, Igor Krupa

**Affiliations:** 1 Center for Advanced Materials, Qatar University, P. O. Box 2713, Doha, Qatar; 2 Centre of Polymer Systems, University Institute, Tomas Bata University in Zlin, Trida T. Bati 5678, 760 01 Zlin, Czech Republic; 3 University Paris Est, CNRS, UMR7182, ICMPE, UPEC, F-94320 Thais, France; 4 QAPCO Polymer Chair, Center for Advanced Materials, Qatar University, P.O. Box 2713 Doha, Qatar

## Abstract

Epoxy polymer nanocomposites filled with magnetite (Fe_3_O_4_) clay (B), named (B-DPA-PANI@Fe_3_O_4_) have been prepared at different filler loading (0.1, 0.5, 1, 3, 5 wt. %). The surface modification of clay by polyaniline (PANI) is achieved in the presence of 4-diphenylamine diazonium salt (DPA). The effects of the nanofiller loading on Tensile, mechanical and dielectric properties were systematically studied. Improved properties was highlighted for all reinforced samples. The addition of only 3 wt. % of the filler enhanced the tensile strength of the composites by 256%, and the glass transition temperature Tg by 37%. The dielectric spectra over a broad frequency showed a robust interface between the hybrid (B-DPA-PANI@Fe_3_O_4_) fillers and epoxy matrix. The results showed most significant improvement in corrosion inhibition using electrochemical impedance spectroscopy (EIS) in 3.5 wt % NaCl, as well as a significant response in oil sensing test. High charge transfer resistance of 110 × 10^6^ Ω.cm^2^ using 3-wt % of filler was noted compared to 0.35 × 10^6^ Ω.cm^2^ for the pure epoxy. The results obtained herein will open new routes for the preparation of efficient anticorrosion sensor coatings.

## Introduction

Nowadays, Intensive research was devoted to design a smart and intelligent multifunctional hybrid polymer nanocomposite materials for emerging applications^1,2^. Particularly, hybrid bio-based materials, in contrast to fossil resources have received much attention^3,4^. they can be produced from many renewable sources. Multi-functionality may be added to those materials collected from renewable sources by combining them with many different materials to achieve the desired functionality^5,6^. such multifunctional bio-based nanocomposite fabrication may involve the incorporation of inorganic component and can produce a product with useful electrical, mechanical, magnetic, and a wide range of applications^7,8^.

Clay, particularly bentonite, is a naturally abundant resource and environmentally friendly due to its biodegradable and renewable features^9^. Moreover, clay is well known to add interesting properties to a polymer matrix, such as flame retardancy^10^, and high storage modulus^11,12^. Bentonite is a swellable clay; it is composed of thin aluminosilicate layers^13^, is hydrophobic in nature. Surface pre-modification is a main key to designing new materials from bentonite. There are various processes of modifications; Using silane-coupling agents^14^, mediating the hydroxyl groups of bentonite located on the sheet, the cation exchange method^15^, or, more recently, the covalent modification using diazonium salts^16^, which produces new interfaces between clay filler and different polymers like poly(methacrylates)^17^ and conductive polymers^18^. Among those polymers, polyaniline is well known as one of the best conducting polymers due to its easy preparation, important electrical and sensing properties^19,20^.

Polyaniline has been used for different sensor applications such as gas, volatile organic compound, pressure and strain^15,21^. However, the weak mechanical properties and poor solubility of PANI limited its commercial uses and experimental studies. Thus, attention has been given to the immobilization of PANI on a variety of materials namely Clays materials, in order to enhance its applicability^20,22^. The alliance of modified clays with polyaniline^23^, improve mechanical^24^, dielectric^25^, magnetic properties^26^, and may offer some added value and applications to the final clay-PANI composites, especially in anti-corrosive coatings^27–30^, and oil sensing applications^31,32^.

On the one hand, metal corrosion is one of the most severe problems in industries^30,33^. Barrier protective coatings^34–36^ (*e.g*. paints) provide an interesting approach to protect against corrosion by using clay-PANI modified with metal oxides^37,38^, as functional additives in which they act as a barrier for moisture or oxygen transportation pathways. Clay-PANI composites have already proved to be anti-corrosive and have become natural candidates for further research^39^. Moreover, the redox behavior of PANI provided self-healing properties to the intentionally scratched coatings^40^. Furthermore, synthesis of epoxy-doped Clay-PANI nanocomposites with different metal oxide nanoparticles such as ZnO^41,42^, TiO_2_^43^, SiO_2_^44^, Fe_2_O_3_^45,46^, ZrO_2_^47^ and Al_2_O_3_^48^ was found to improve the corrosion protection of carbon steel via the adjustment of the interaction between the clay-PANI nanocomposites and the added metal oxide Nano-species^49–51^. The most discussed mechanism of PANI based nanocomposites in the literature is the called “ennobling mechanism”^52^. It is focused on the assumption that the conductive polymer acts as an oxidant and maintains the metal in the passivity domain. This mechanism could induce the oxidation of the free metal surface at small defects in the passive layer^53^.

On the other hand, oil is considered as main reasons of water contamination, particularly in marine environment^54^. Oil spills could be caused by the release of crude oil from offshore platforms^55^, by derivate products used by huge ships^56^.

Several set up controlling oil pollution are available, and they mostly measure specific properties such as light scattering, fluorescence, and so on. These existing devices are usually huge (e.g. require water to be pumped in), expensive and consume significant amounts of energy^57^.

Therefore, significant research work has been dedicated to designing new, low-cost, smart sensors^2^ that can be directly used, in order to provide quick and quantitative evidence about organic contaminants in biosphere. Indeed new hybrid materials based on conductive polymers, namely PANI have been developed^58,59^. The conductive polymers provide an appropriate level of electrical conductivity of the material at low concentration. The operation of this sensor is based on the electrical resistance changes of the hybrid composites when exposed to oil.

In this way, we sought to design new hybrid and functional material (relevant to corrosion protection and oil sensing) by utilizing a naturally abundant material (bentonite) as active diazonium modified platform for the immobilization of the prepared DPA-PANI@Fe_3_O_4_ magnetite hybrid filler. DGEBA epoxy resins are selected as a matrix for blending the prepared hybrid filler, as it is the most widely used thermosetting resin. It is very well documented that the addition of well-dispersed fillers into DGEBA epoxy resin can significantly increase the mechanical^60^, thermal^61^, anticorrosion^62^, and other important properties^63^. The surface modification of the clay with polyaniline (PANI) was achieved using an *in-situ* surface-initiated polymerization method in the presence of grafted diazonium salts to the bentonite surface to provide well-dispersed epoxy nanocomposites. The loading effects of the prepared filler were studied. The thermal stability of the filled epoxy was studied by thermogravimetric analysis (TGA) in addition to mechanical properties such as dynamic mechanical (DMA) and tensile analysis. Interface studies between the prepared filler and the epoxy matrix were investigated using the dielectric properties. The fracture surface of the cured and filled epoxy was observed by scanning electron microscope (SEM). Finally the DGEBA matrix filled B-DPA-PANI@Fe_3_O_4_, was tested simultaneously as oil sensor and anti-corrosion coating in 3.5 wt % NaCl media.

To the best of our knowledge, such an investigation using B-DPA-PANI@Fe_3_O_4_ nanohybrids, designed by the reaction of bentonite through the *in situ* polymerization of aniline in the presence of 4-diphenylamine diazonium salt, for smart anticorrosion sensors, has not been previously reported. That was the motivation for this project.

## Experimental

The DGEBA (Bisphenol A diglycidyl ether), the 4,4′-diaminodiphenylsulfone (DDS) were purchased from Sigma-Aldrich. Bentonite was purified according to a standard procedure^64^ resulting in ∼80-μm-sized bentonite (B). The cationic exchange capacity (CEC) was equal to 101.9 meq/(100 g of clay). Fe_3_O_4_ nanopowder (Sigma Aldrich, 97% purity, 50–100 nm), N-phenyl-p-phenylenediamine (Acros, 98% purity), isopentyl nitrite (Alfa Aesar, purity 97%), ammonium persulfate (APS, Acros, 98% purity), and nitric acid (Carlo Erba, 60% purity). Aniline (Aldrich, 99.5% pure) was purified and stored at low temperature before usage. Distillated water for cleaning and dilutions was used throughout.

## Synthesis of the Hybrid Filler B-DPA-PANI@Fe_3_O_4_

The B-DPA-PANI nanocomposites clay/polyaniline were prepared as function of the cation exchange capacity (CEC)^18^ by polymerization of aniline on the 4-diphenylamino diazonium-exchanged clay as active platform. The B-DPA-PANI@Fe_3_O_4_ hybrid magnetite filler were prepared with reference to mechanochemical synthesis process^65^. B-DPA-PANI were used as starting materiel and Fe_3_O_4_ nanoparticles (Sigma Aldrich) as magnetite substrate. Samples were mixed at a (in a 1:1 weight percent ratio) using a Retsch PM400 planetary ball mill, with the milling speed of 450 rpm in order to get a homogeneous particle distribution for 2 h and in the two-step milling operation^65^.

## Preparation of the Composites

### Preparation of B-DPA-PANI@Fe_3_O_4_/DGEBA Resin Suspensions

DGEBA resin suspensions containing (0.1, 0.5, 1, 3, 5 wt. %) of the as-prepared hybrid filler were prepared. Fillers in different ratios were mixed with the DGEBA epoxy resin and sonicated (probe sonicator) for 10 min before being mechanically stirred for one hour.

### Curing of the B-DPA-PANI@Fe_3_O_4_/DGEBA Resin

The DDS hardener was added into the B-DPA-PANI@Fe_3_O_4_ (0.1, 0.5,1, 3, 5 wt. %)/DGEBA resin suspensions at 180 °C with vigorous stirring; it was then poured into a metallic mold (12 cm × 15 cm × 3 mm thick), cured for 4 h at 180 °C, post cured at 200 °C for 1 h and cooled naturally to room temperature.

### Preparation of coatings for corrosion, dielectric and oil sensing study study

B-DPA-PANI@Fe_3_O_4_/DGEBA resin coatings were prepared using probe sonicator (UP 400 S ultrasonic processor) by dispersing (1, 3, 5 wt. %) of B-DPA-PANI@Fe_3_O_4_ fillers in DGEBA epoxy resins. The DDS curing agent was then added to the mixture. The application of coating was accomplished by using a doctor blade (500 mm).

### Characterization

TGA measurements were accomplished under nitrogen using a TGA 4000 (Perkin Elmer, USA) from 30 °C to 700 °C (heating rate of 10 °C/min). The X-ray diffraction (XRD) measurements were performed using PANalytical instrument (modelX’PertPRO) with Co Kα (1.789 A°) radiation. Tensile properties were tested by using a LIoyd LR50K-Plus universal testing machine (UTM), equipped with a 10 kN load cell at a displacement rate of 5 mm/min at room temperature as per ASTMD 638. Flexural properties were determined using rectangular bars having dimensions of 127 mm × 12.5 mm × 4 mm on the same machine, at a speed of 10 mm/min as per ASTM D 790. The fracture surfaces of the samples were studied using a Nova Nano SEM 450 Scanning Electron Microscope.

Dynamic mechanical analyses were conducted using a RSA-G2 (TA Instruments, USA) in 3-point bending mode in the linear viscoelastic region (LVR). Rectangular samples (40 mm × 8 mm × 1.2 mm) were prepared and investigated from 30–250 °C (3 °C/min heating rate), with a strain deformation of 0.007% and a frequency of 1 Hz. The thermal stability of the samples was analyzed using a TGA Pyris 4000 from 30–800 °C (heating rate of 10 °C/min). Dielectric measurements were performed using a Novocontrol GmbH Concept 40 broadband dielectric spectrometer (Montabaur, Germany), and data were collected at room temperature over the frequency range of 0.01 Hz–2 MHz. Sample discs (2 cm diameter) were sandwiched between two gold-coated copper electrodes (2 cm diameter) and transferred to the instrument for data collection.

Experimental data were described using Havriliak-Negami model equation 1^5^,

1 $${\varepsilon }_{HN}^{\ast }(\omega )={\varepsilon ^{\prime} }_{\infty }+\frac{{\rm{\Delta }}\varepsilon ^{\prime} }{{(1+{(i\omega \cdot {t}_{rel})}^{a})}^{b}}$$

where Δε′ = ε′s − ε′∞ is the dielectric relaxation strength; ε′s and ε′∞ are relative permittivities at zero and infinite frequencies, *f*, respectively; *ω*, is angular frequency (=2 π *f*); *t*rel is the relaxation time; and a and b are shape parameters describing the asymmetry of the dielectric function. Electrochemical impedance spectroscopy (EIS) measurements were performed in a NaCl solution (3.5 wt. %) in a frequency range of 0.01 Hz to 100 kHz with a wave amplitude of 5 mV at 25 °C. EIS data analysis was performed using Gamry Echem analyst software. Before conducting the EIS experiments, samples were immersed in the 3.5 wt. % NaCl electrolyte for 30 min. The water contact angles (WCA) of the fabricated coatings were measured using Dataphysics (OCA 35, Germany) with 5 μL distilled water.

The oil sensing experiments were conducted by measuring the electrical conductivity of sample using a Novocontrol GmbH Concept 40 broadband dielectric spectrometer (Montabaur, Germany). The electrodes were coated by using silver paste on the surface of the sample and maintaining a prescribed distance (1 mm). The oil drop was applied to the other side of the electrode to avoid oil and electrode interactions.

## Results and Discussion

### Effect of Diazonium Cation and Fe_3_O_4_ Intercalation on clay/Polyaniline Properties

The B-DPA-PANI conductive hybrid fillers were initially prepared by the covalent bonding of the diazonium cation to bentonite surface, followed by the oxidative polymerization of the aniline monomer as formerly reported^24^. Then the B-DPA-PANI@Fe_3_O_4_ hybrid magnetite filler were prepared with reference to mechanochemical synthesis process^65^ in order to get a homogeneous particle distribution inside clay galleries.

**Figure 1:**
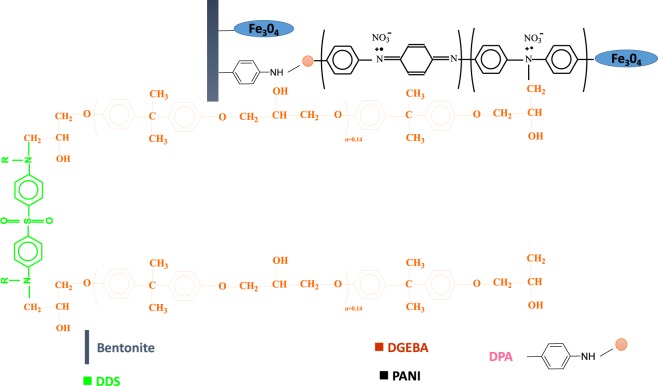
Molecular view of the DGEBA- B-DPA-PANI@Fe_3_O_4_ interface.

Figure 1 displays the interface chemistry of the as prepared magnetite hybrid nanofiller with the DGEBA epoxy resin and the DDS hardener. The as prepared hybrid magnetite filler has highly dispersed and stable Fe_3_O_4_ nanoparticles, NH groups from both DPA and PANI, which could react with epoxy groups via ring opening, resulting in covalent bonding of the resin to the clay sheets (via PANI and DPA). Moreover, The DDS hardener has two amino groups, which may react with epoxy by the same mechanism.

Table 1 summarize the most important properties of the prepared hybrid materials. The introduction of the diazonium salt and Fe_3_O_4_ are important to prepare a new conductive and exfoliated hybrid filler.

**Table 1:** Summary of Preparation Methods as well as Electrical and Morphological Features of Clay/PANI Nanocomposites.

	B-/PANI^18^	B-DPA/PANI@Fe_3_O_4_
Surface modifier		4-diphenylamine diazonium salt (DPA) +Fe_3_O_4_
Experimental details	Oxidative polymerization of aniline in presence of purified clay	Purified bentonite (B) first covalently bonded to the DPA, followed by the oxidative polymerization of aniline
Structure and crystallinity	same basal distance as in purified clay(1.38 nm)	-exfoliated bentonite structure -crystalline structure of Pani -crystalline structure of Fe_3_O_4_
Conductivity S·cm^−1^	σ = 2.1 × 10^−8^	σ = 3.4 × 10^−2^

Hereafter, we will report the impact these new fillers had on Interfacial, morphology, mechanical, tensile, and dielectric properties of DGEBA epoxy resins as well as its potential application in corrosion protection and oil sensing.

### IR and XRD of the prepared nanofillers

**Figure 2:**
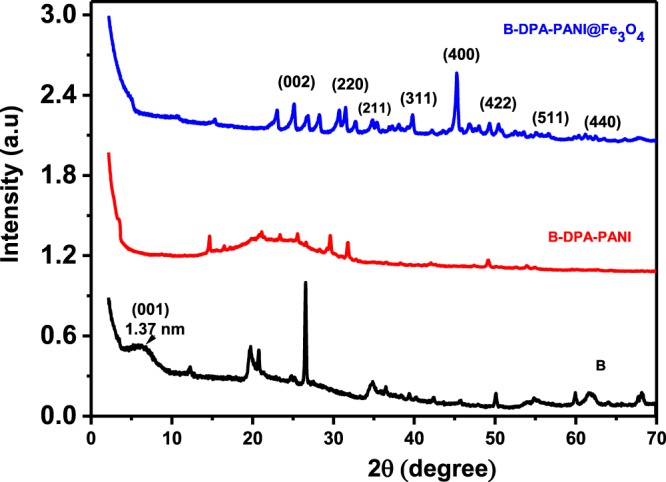
XRD patterns of B, B-DPA-PANI and B-DPA-PANI@Fe_3_O_4_.

The XRD patterns of the purified bentonite B, prepared B-DPA-PANI, and B-DPA-PANI@Fe_3_O_4_ nanocomposites are shown in Fig. 2. Bentonite is characterized by a diffraction peak at 2 ϴ = 6.67 which corresponds to an interlayer distance equal to 1.37 nm; this diffraction peak disappeared for the B-DPA-PANI and DPA-PANI@Fe_3_O_4_ nanocomposites and confirms the exfoliation of the bentonite after the polymerization of aniline in the presence of the diazonium (DPA) coupling agent. Moreover, the broad peaks at 19–20° and 25–26° confirmed the grafting of PANI chains to the bentonite sheets, corresponding to the (020) and (200) reflections of the emeraldine PANI salt^66^. For the B-DPA-PANI@Fe_3_O_4_, diffraction peaks appeared at ∼30°, 35°, 43°, 53°, 57° and 62°, which may be assigned to (220), (311), (400), (422), (511) and (440), respectively—the inverse spinel phase of Fe_3_O_4_ (JCPDS 01-1111).

### Microstructure of fractured surface of the nanofiller filled epoxy

To confirm the presence of the prepared filler in the epoxy composites, SEM images in the mapping mode were obtained (Fig. SI2). The red colored individual particles contain the iron structure; clearly showing that the particles are homogenously dispersed within the cured DGEBA and can improve the physical characteristics of the prepared composites.

**Figure 3:**
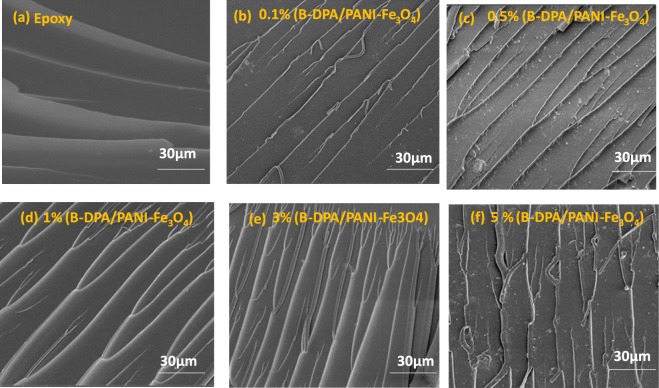
SEM images of the fracture surfaces taken from tensile specimens of cured pure DGEBA, 0.1, 0.5, 1, 3 and 5-wt % B-DPA/PANI-Fe_3_O_4_ filled DGEBA.

Figure 3 displays fracture surfaces for untreated and DGEBA with different weight loadings of B-DPA/PANI-Fe_3_O_4_. Figure 3a shows a smooth fracture surface together with the river-like structure^67^. However, radical change was observed in the morphology of the blended DGEBA. Figure 3(b–e) reveal the formation of a strong network microstructure within the DGEBA resin. This unusual morphology is most likely induced by a very strong filler−matrix (hybrid magnetite filler-DGEBA) adhesion. However, for the 5% added nanofiller, there is showed in Fig. 3(f) some cleavage in the fibril network structure to a small broken segments was observed. This could be ascribed to the high number of crosslinks present in the cured DGEBA. Indeed, PANI can act also as a (secondary) cross linker in addition of the DDS (principal hardener). This latter is most likely the cause of the agglomeration of the used filler, hence the fragility and breaking of the fibril microstructure.

### Mechanical Properties

The mechanical behavior of the composites in the tensile mode was investigated.

**Figure 4:**
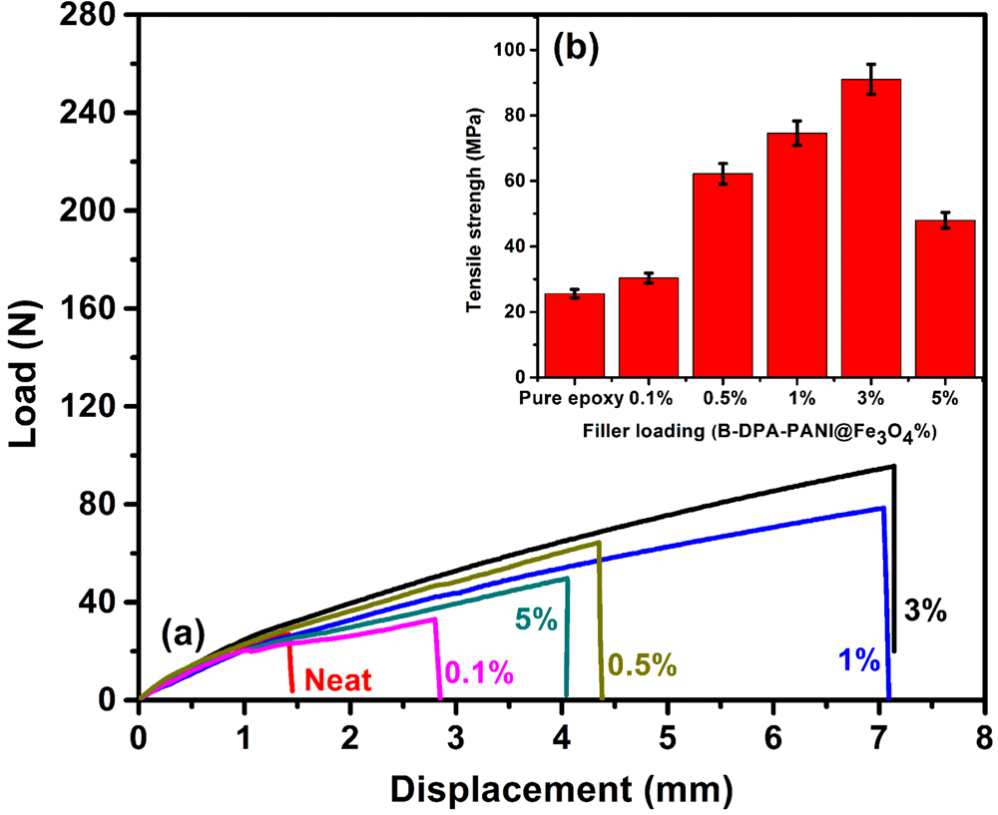
Tensile load−displacement curves of the cured epoxy and epoxy nanocomposites filled with different nanofiller loadings (0.1, 0.5, 1, 3 and 5.wt %).

The tensile strength of epoxy containing various B-DPA/PANI filler loadings is shown in Fig. 4. Addition of low nanofiller loadings (0.1. 0.5, 1 and 3% wt.) showed significant enhancements in the tensile strength of the epoxy nanocomposites (~23%, 143%, 206% and ~256%, respectively). This finding could be due to efficient dispersion of the nanofiller as well as robust filler-matrix physico-chemical interfaces achieved between the particles and the epoxy matrix^24^, which not only increases the epoxy monomer dispersion over faster intralamellar reaction but also reacts with epoxy chains. This reinforcing mechanism will lead to an increase in the strengths of the epoxy nanocomposites^68^. This can be likely due to the very well dispersion of nanofiller (0.1–3 wt %) loading, resulting in the strong interface between the hybrid magnetite filler and the DGEBA resin. Nevertheless, the observed lower tensile with 5wt % of nanofiller, can be caused by the magnetite filler agglomeration as described previously from SEM micro-structure.

### Viscoelastic Properties of Filled Epoxy by DMA

**Figure 5:**
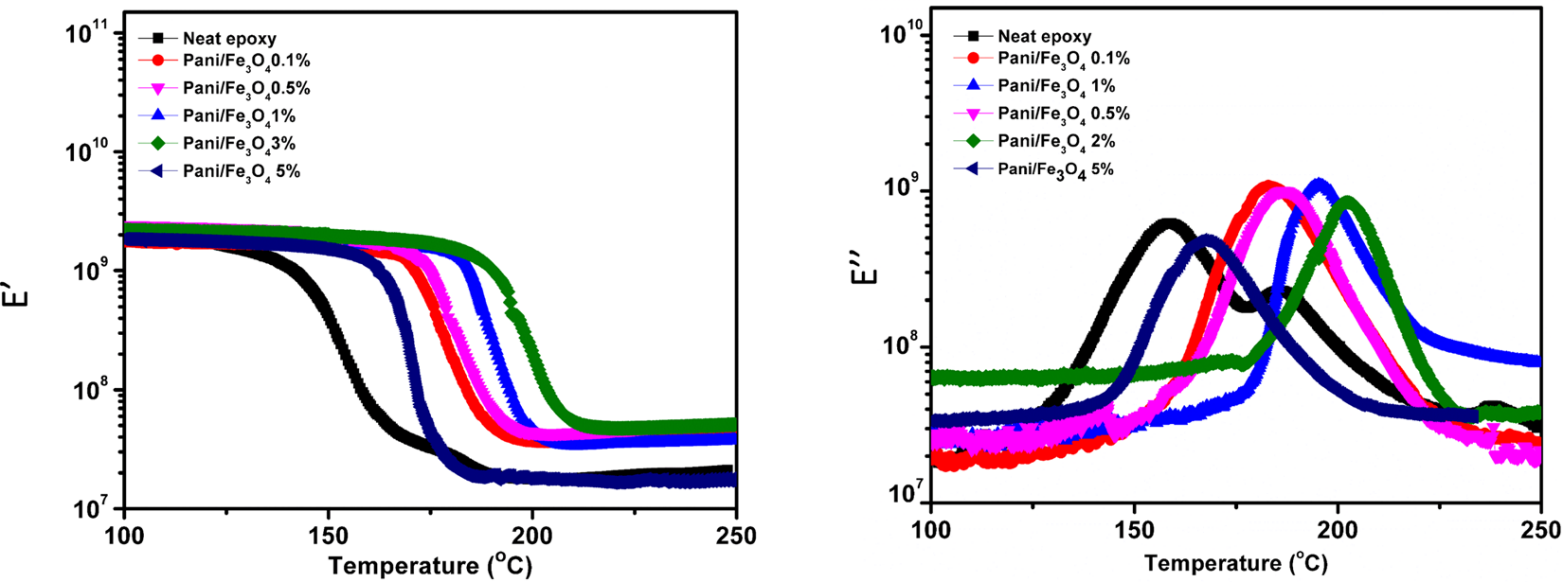
Temperature dependence of storage (right) and loss (left) moduli for the cured pure epoxy and filled epoxies with 0.1, 0.5, 1, 3 and 5 wt. % B-DPA/PANI-Fe_3_O_4_ loadings.

Dynamic mechanical analyses provide evidence on the incorporation of the hybrid filler in the epoxy matrix as well as of its exfoliation via a mechanical performance investigation. In Fig. 5, the glass transition temperature (Tg) of the neat matrix is close to that observed for the 0.1 wt. % composite hybrid filler. Moreover, the Tg increased as the amount of hybrid filler increased in the epoxy matrix and shifted from 150 °C to 205 °C; this shift represents a significant enhancement. In general, exfoliation of the hybrid filler in the matrix increases with homogenous filler dispersion and is confirmed by XRD and SEM mapping. The system increased the Tg due to the polymer chain mobility restrictions and sufficiently raised toughness as evidenced by DMA investigation. However, for 5wt % composite hybrid filler, there is a radically decrease of the (Tg), most likely due to the agglomeration of the magnetite hybrid filler in the DGEBA resin. This might be attributed to the high density of crosslinks present in the blended DGEBA: Resulting in the agglomeration of the filler, thus the decrease of mechanical properties, confirmed in the previous section by the SEM microstructure and tensile strength properties.

### Dielectric Properties of Composites

**Figure 6:**
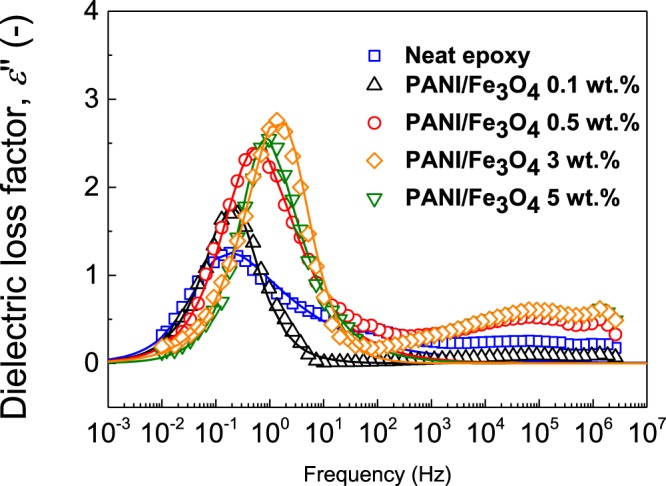
Dielectric spectra in the broad frequency range for the cured pure epoxy and filled epoxy with 0.1, 0.5, 3 and 5-wt % B-DPA/PANI-Fe_3_O_4_ loadings.

To investigate the interfacial processes in the epoxy-based composites, the room temperature dielectric spectra over a broad frequency range were evaluated. The experimental data were then fit with Havriliak-Negami model, and parameter identification provided us with information on how the hybrid filler influenced the epoxy composites. Generally, in the composite systems, the Wagner-Maxwell-Sillars (MWS) relaxation occurs at low frequencies. The interfacial processes are intensified because of the hybrid filler addition. As seen in Fig. 6, the peak maxima increase as the amount of filler increases up to 3 wt%. In addition, the relaxation time (Table 2) as a measure of the process activity decreases, indicating a strong interaction between the hybrid filler and matrix. From Table 2, it can also be seen that relative permittivity extrapolated to zero frequency increases as the filler content increases, which confirms the enhanced dielectric properties. These results provide clear evidence of improved properties due to the enhanced filler-matrix interaction up to 3 wt% and is in good agreement with the results obtained from DMA, tensile strength and microstructure investigations.

**Table 2:** Parameters of the modified Havriliak-Negami model (eq. 1) for the various epoxy composites.

	Neat Epoxy	0.1%	0.5%	3%	5%
*ε*″_∞_	2.09	2.26	2.02	2.71	3.16
Δ*ε′*	4.96	3.77	7.09	6.69	6.12
*t*_rel_ [s]	2.31	0.72	0.41	0.07	0.25
*a*	0.89	0.93	0.87	0.79	1
0.98	0.37	1.15	0.68	1.53	0.68
*ε′*_s_	6.05	7.03	9.11	9.4	9.28

### TGA of the nanofiller filled epoxy

The TGA spectra showed in SI1 that the addition of the hybrid filler does not affect the properties of the composites and is most likely due to the low filler loading. Even if the thermal properties were not significantly enhanced, the improvement imparted in mechanical and dielectric properties is appreciable.

### Anticorrosion performance

**Figure 7:**
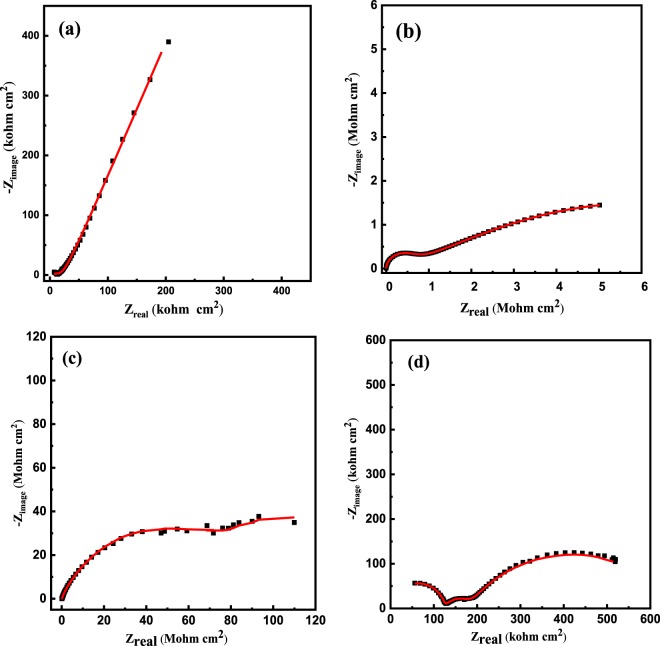
Nyquist (**a**–**d**) plots of epoxy coatings with 0 (**a**), 1 (**b**), 3 (**c**) and 5 (**d**) wt. % BP-DPA-PANI@Fe_3_O_4_ polymer.

**Figure 8:**
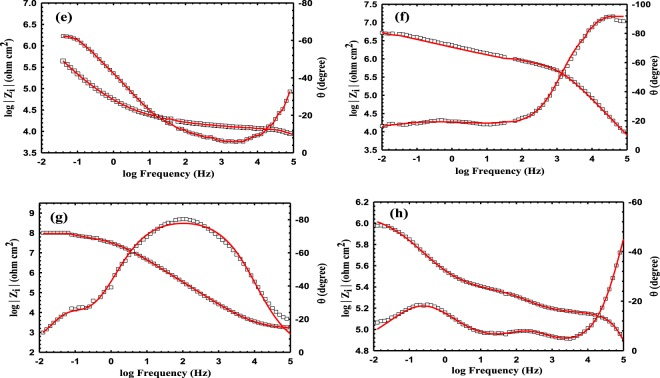
Bode (**e**–**h**) plots of epoxy coatings with 0 (**e**), 1 (**f**), 3 (**g**) and 5 (**h**) wt. % BP-DPA-PANI@Fe_3_O_4_ polymer.

**Figure 9:**
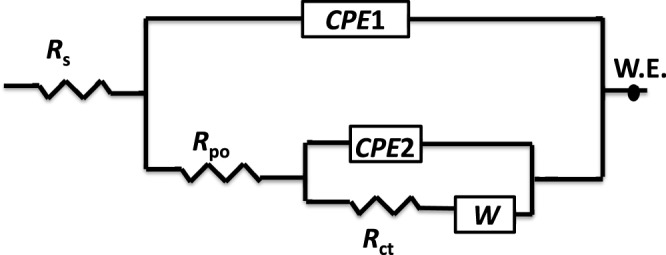
Equivalent electrical circuit used to fit the EIS spectra of the different nanocomposite coatings in seawater.

Electrochemical impedance spectroscopy (EIS) experiments are conducted to explore how the magnetite polymer in the epoxy coatings influences their corrosion protection efficiency. Figures 7 and 8 show the Nyquist and the Bode plots of the nanocomposites in 3.5 wt. % NaCl, respectively. The measured EIS data are presented by the scattered symbols, and their fitted lines are the solid ones. Fitting is done using the equivalent circuit (Fig. 9).Three measurements were conducted out for all of the as-prepared coatings. The reproducibility of the results was good.

The EIS parameters, derived from fitting the EIS data using the equivalent circuit shown in Fig. 9, are listed in Table 3.

**Table 3:** Corrosion parameters obtained from the EIS data for the corrosion of pure epoxy containing different concentrations of BP-DPA-PANI-Fe_3_O_4_.

The wt. % of the filler	*R*_ct_ Ω.cm^2^	*R*_po_ Ω.cm^2^	*CPE*1 μF cm^−2^ s^α−1^	*n*_1_	*C*_coat_ μF cm^−2^	*CPE*2 μF cm^−2^ s^α−1^	*n*_2_	*C*_dl_, μF	*W*, Ω.cm^2^ s^−1/2^	Goodness of fit
0	0.35 × 10^6^	0.2 × 10^6^	13	0.876	14	23	0.828	35	89 × 10^−6^	1 × 10^−4^
1	9.8 × 10^6^	7 × 10^6^	0.7	0.813	1	2	0.798	4	0.62 × 10^−6^	2.2 × 10^−4^
3	110 × 10^6^	53 × 10^6^	0.06	0.782	0.08	0.1	0.773	0.4	0.03 × 10^−6^	3.2 × 10^−4^
5	0.6 × 10^6^	0.5 × 10^6^	5	0.811	6	8	0.769	12	31 × 10^−6^	2.4 × 10^−6^

*R*_s_, *R*_po_ and *R*_ct_ are the solution, pore and charge transfer resistances, respectively. In addition, CPE and *W* are the constant phase element and Warburg impedance, respectively. The impedance of the CPE is calculated using the formula 1/Z_CPE_ = *Q*^o^ (j*w*)^α^, where *Q*° (s. Ω^−1^) equals the admittance (1/|*Z*|) at *ω* = 1 rad/s, *ω* is the angular frequency of the AC signal (1/rad) and *α* is the CPE exponent^69–76^. As *α* approaches 1, the CPE behavior approaches ideal capacitor behavior. It is worth mentioning that both of CPE1 and CPE2 were used instead of a regular capacitor element, to estimate the value of the coating capacitance (C_c_) and the double layer capacitance (*C*_dl_); using the following formula^77,78^.

$${C}_{dl}=\sqrt[n]{\tfrac{{Q}}{{R}_{x}(\alpha -1)}}$$

where, Q is CPE constant, α is CPE exponent, respectively. *R*_x_ represent the pore resistance (*R*_po_), or the charge transfer resistance (*R*_ct_).

Good fittings were obtained with Chi-square (Χ^2^) using the equivalent circuit in Fig. 9, see Table 3. The magnitude of impedance modulus at low frequency (|*Z*|_0.01_ Hz), is an suitable element for calculating the overall corrosion protection efficiency of the as-prepared coatings, while the charge transfer resistance (*R*_ct_) reflects the resistance to electron transfer across the metal solution interface underneath the coating which is inversely proportional to the undercoating corrosion rate.

The high impedance values ~10^6^ Ω cm^2^ at the low frequency region in the EIS measurements confirms the good corrosion protection efficiency of the nanocomposite coating. As the wt. % of the magnetite polymer increases, the *R*_ct_ and *R*_po_ increase from 0.35 × 10^6^ Ω cm^2^ and 0.2 × 10^6^ Ω cm^2^ for pure epoxy to 110 × 10^6^ and 53 × 10^6^ Ω cm^2^ respectively, after the addition of 3 wt. % of the filler. However, at a higher content of the filler (5 wt. %), the *R*_ct_ and *R*_po_ decrease significantly to 0.6 × 10^6^ and 0.5 × 10^6^ Ω cm^2^, respectively. In addition, the double layer capacitance at the coating/metal interface distinctly decreased from 35 µF for the pure epoxy to 0.4 µF for the 3 wt. % composite of the magnetite polymer, and the Warburg coefficient consequently decreased from 89 × 10^−6^ for the pure epoxy to 0.03 × 10^−6^ Ω cm^2^ s^−1/2^ for the 3 wt.% nanocomposite. Many reports have suggested different mechanisms of the corrosion protection of the doped PANI with an epoxy coating especially when a low content of PANI is used. Ramezanzadeh *et al*.^79^ found that the addition of graphene oxide/polyaniline (GO-PANI) to a zinc-rich epoxy increases the protection efficiency of the carbon steel because the deposited PANI existed in the emeraldine salt (PANI-ES) form, which was converted to emeraldine base (PANI-EB) by capturing the released zinc particles from the corrosion process. However, in the presence of Cl^−^ ions, the PANI-EB was reconverted to PANI-ES and completed the autocatalytic cycle, which stabilized the Fe in the passive region and Zn in its active form. Kinlen *et al*.^80^ used the scanning reference electrode technique (SRET) to prove that polyaniline (PANI) passivates pinhole defects that exist in the coating matrix on carbon steel. On the other hand, Hosseini *et al*.^81^ attributed the corrosion protection of their nanocomposite coating (EP/DBSA doped PANI-TiO_2_) to the titania nanoparticles as an inert material in addition to the produced Fe_2_O_3_ at the coating/metal interface that fills the pores of the coating matrix which hindered the attack of the corrosive ions. In addition to the aforementioned reasons for the corrosion resistance of PANI, it is observed that the filler used in this study decreases the hydrophilicity of the as-prepared coatings up to a maximum amount of filler, after which the hydrophilicity increases again which lowers the corrosion resistance.

**Figure 10:**
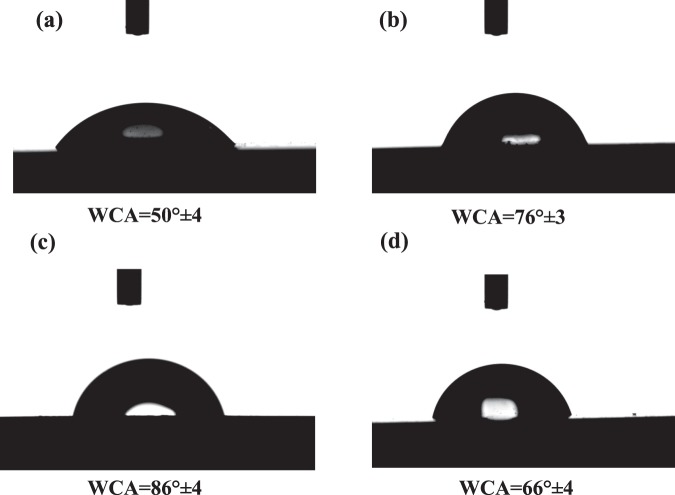
The measured water contact angle of before addition of the filler (**a**) pure epoxy coating and after the addition of the magnetite polymer (**b**) 1 wt. %, (**c**) 3 wt. % and (**d**) 5 wt. % of B-DPA/PANI- Fe_3_O_4_.

The corrosion protection of the magnetite polymer can be attributed to that filler which decreases the porosity of the nanocomposite coating and therefore decreases the diffusion of the chloride ions inside along with the corrosion products out of the coating^82^. However, a further increase in the concentration of the magnetite nanoparticles (>3 wt.%), leads to a noticeable decrease in the corrosion protection of C-steel, which could be attributed to the agglomeration of the magnetite nanoparticles particles^83^, that leaves defects (such as pinholes or pores) in the coating matrix and increases the diffusion of the aggressive ions through the coating^81^. The agglomeration is produced by both van der Waals forces and electrostatic attraction of the charges that exist on the magnetite nanoparticles surface^84^. On the other hand, increasing the content of the hydrophobic PANI in the epoxy coating decreased the hydrophilicity of the nanocomposites and consequently increased the WCA from 50° ± 4 to 85° ± 2 for the 3 wt.% of BP-DPA-PANI-Fe_3_O_4_ (Fig. 10), which resulted in a decrease in the diffusion of the ions through the coating. However, further addition of the filler (5 wt. %) decreased the WCA to 75° ± 3 and resulted in a significant decrease in the corrosion resistance. The decrease in the hydrophilicity after the addition of the filler could be attributed to the increase of the surface roughness as shown in SI3. Nevertheless, at 5 wt.% the surface roughness prominently decreased to 10 nm due to agglomeration and non-homogeneous distribution of the nanoparticles.

### Oil sensing application

**Figure 11:**
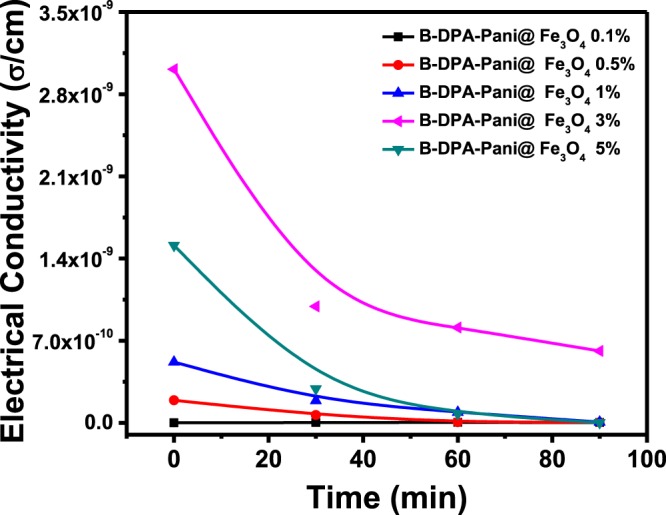
Sensing characterization of samples. Conductivity (Ϭ/cm) vs time (min) for DGEBA-B-DPA-PANI@Fe_3_O_4_ composite films in oil.

It has been described that conducting polymers composites such as PANI and polypyrrole are being used as sensitive materials for oil sensing applications due to their inexpensive and facile room temperature preparation^85^. In this work, the sensitivity of the composites’ sensing property was measured by connecting both the ends of the filmstrip and dipping it in oil. One emerging application of these composite films is as an oil sensor to detect oil on the surface of marine vehicles. It is also popularly utilized as an anticorrosive coating. All sensing experiments fundamentally rely on the measurement of electrical conductivity of the developed samples. In the previous reports, as well as in our study, it acts as elementary principle behind all sensing^86,87^. The chemical nature of the matrix is by far the most vital parameter for sensing and influences the electrical properties aside from other variables, such as the nature of the polymer matrix, filler concentration and resistance^88^. As seen in Fig. 11, measurements for conductivity were performed in an oil media at 25 °C for all samples. The conductivity measurements followed a decreasing trend for the samples, which is a rare and unlikely change. The highest conductivity change in the oil occurred with epoxy filled with 3 wt. % of B-DPA-PANI@Fe_3_O_4_ fillers, at 5 wt% the conductivity start to decrease due magnetite hybrid filler agglomeration as described previously. The explanation behind this kind of conductivity behavior can be attributed to the absorbance and expansion of some molecules at the time of oil exposure. That expansion leads to a decrease in the effective filler volume fraction, which in turn decreases the overall electrical conductivity.

## Conclusions

In this work, we have shown that the surface chemistry of aryl diazonium salts combined with magnetite nanoparticles is versatile and can be efficiently employed to modify the surface of natural bentonite. It permits the chemical binding with bentonite surface from one side; moreover, it provides anchoring sites for the *in situ* polymerization of aniline resulting in a new hybrid material, which exhibits a polymer rich surface with unique properties. The later was used as filler for DGEBA at different weight loading and was found to improve interfacial, hydrophobicity, mechanical and dielectric conductivities of the epoxy resin for all reinforced samples up to 3 wt%. All results confirmed a strong interaction between the hybrid magnetite filler and DGEBA. Moreover, the ability of BP-DPA-PANI@Fe_3_O_4_ nanocomposite film as a protective layer to prevent corrosion of carbon steel as well as oil sensor has been studied. Results confirmed that the prepared nanocomposites supply protection for the carbon steel, the highest charge transfer resistance of 110 × 106 Ω.cm^2^ was achieved using 3 wt.% only of the prepared filler. It was found that the specific electrical conductivity of the materials (Ϭ/cm) strongly depends on to the absorbance and expansion of some molecules at the time of oil exposure, highest conductivity change in the oil occurred with epoxy filled with 3 wt. % of magnetite filler.

From the above, this approach clearly highlights a new surface and interface chemistry using diazonium salt to prepare efficient and inexpensive bio-based epoxy, for oil sensing & anti-corrosive smart protection with very strong interfacial interactions between filler and host matrix. This strategy can be used potentially to development of smart coatings such as Painting.

## Electronic supplementary material

Supplementary Information
