# Supplementary material for: Anti-corrosive and oil sensitive coatings based on epoxy/polyaniline/magnetite-clay composites through diazonium interfacial chemistry
Source: Sci Rep. 2018 Sep 6;8:13369. doi: 10.1038/s41598-018-31508-0 (PMC6127100; doi:10.1038/s41598-018-31508-0)
Supplement: Supplementary file 1 — Supplementary Information [file 41598_2018_31508_MOESM1_ESM.docx]

**Supplementary information**

**Anti-corrosive and oil sensitive coatings based on epoxy/polyaniline/magnetite-clay composites through diazonium interfacial chemistry**

Khouloud Jlassi^a*^, A. Bahgat Radwan^a^, Kishor Kumar Sadasivuni^a^, Miroslav Mrlik^b^, Aboubakr M. Abdullah^a^, Mohamed M. Chehimi^c^, Igor Krupa ^a,d*^

^a^ Center for Advanced Materials, Qatar University, P. O. Box 2713, Doha, Qatar.

^b^ Centre of Polymer Systems, University Institute, Tomas Bata University in Zlin, Trida T. Bati 5678, 760 01 Zlin, Czech Republic.

^c^ Univ Paris Est, CNRS, UMR7182, ICMPE, UPEC, F-94320 Thais, France.

^d^ QAPCO Polymer Chair, Center for Advanced Materials, Qatar University, P.O. Box 2713, Doha, Qatar.

*corresponding author: [igor.krupa@qu.edu.qa](mailto:igor.krupa@qu.edu.qa), [khouloud.jlassi@qu.edu.qa](mailto:khouloud.jlassi@qu.edu.qa)

**Supporting Information SI1**

**Figure SI1:** TGA of cured epoxy filled with 0.1, 0.5, 1, 2 and 3-wt % of B-DPA-PANI@Fe_3_O_4_ Filler loading.

**Supporting Information SI2**


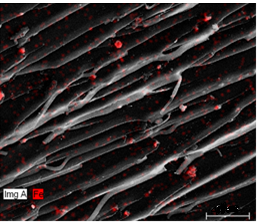


**2 µm**

Figure SI2: SEM mapping images of the fracture surfaces taken from tensile specimens of 3 wt. % BDPA/PANI-Fe_3_O_4_.

**Supporting Information SI3**

**Figure SI3.** The measured value of the surface roughness after addition of different magnetite polymer ratios (a) neat epoxy, (b) 1 wt. %, (c) 3 wt. % and (d) 5 wt. %.
